# Renal cell carcinoma coexistence with ipsilateral renal duplex system: a case report

**DOI:** 10.11604/pamj.2022.42.42.34539

**Published:** 2022-05-16

**Authors:** Rajesh Domakunti, Jay Dharamshi, Abhijit Dhale

**Affiliations:** 1 Department of General Surgery, Acharya Vinoba Bhave Rural Hospital, Datta Meghe Institute of Medical Sciences, Sawangi (Meghe), Wardha, Maharashtra, India,; 2 Department of Urology, Acharya Vinoba Bhave Hospital, Datta Meghe Institute of Medical Sciences, Sawangi, Wardha, Maharashtra, India

**Keywords:** Congenital, bifid ureter, renal cell carcinoma, case report

## Abstract

The duplex kidney is one of the common congenital anomalies of the kidney and urinary tract. Meanwhile, renal cell carcinoma is also the most typical variant constituting more than 90% of cancers of the kidneys. However, renal cell carcinoma is rare in a duplicated collecting system, and literature regarding its association is very sparse. Herein, we present a 28-year-old male patient who presented with abdominal pain and hematuria and was diagnosed with left upper pole renal cell carcinoma coexisted with a duplicated collecting system by computed tomography scan. The patient underwent a left partial nephrectomy followed by meticulous suturing of opened pelvicalyceal system. The histopathology result showed clear cell carcinoma. The postoperative period was uneventful, and the patient was discharged without complications. In conclusion, the coexistence of the duplex system and renal cell carcinoma in the ipsilateral kidney is rare and maybe more than a coincidence, requiring a deeper insight and further elucidation.

## Introduction

Congenital anomalies of the kidney and urinary tract (CAKUT) presents a broad range of anatomical deviations [1]. Various congenital disorders like renal and ureteropelvic anomalies, duplex collecting systems, bladder, and urethra anomalies are included under CAKUT [2]. If CAKUT is one subset of diseases, then Renal Cell Carcinoma (RCC) is an entirely different subset of diseases of the same organ. More than 90% of kidney cancer is constituted by RCC, in which the origin of cancer is from the renal epithelium [3]. Nevertheless, the incidence of both disease entities in the same kidney is rarely documented in the English literature, with sparse knowledge regarding aetiopathogenesis [2,4]. Herein, we reported a 28-year-old man admitted to our clinic with simultaneous RCC and duplex renal pelvis with bifid ureters of the left kidney.

## Patient and observation

**Patient information:** a 28-year-old man, literate, presented to the uro-surgery outpatient department of Acharya Vinobha Bhave Rural Hospital with chief complaints of pain in the abdomen for two months and hematuria for three days. The pain was a dull aching type of mild intensity, non-radiating and intermittent in nature, with no aggravating or relieving factors associated with it. There is no existing co-morbidities or addictions in the patient. No significant past surgical history or family history was noted.

**Clinical findings:** regarding general physical examination, the patient had no obvious clinical findings with a soft & non-tender abdomen without any palpable lump.

**Figure 1:**
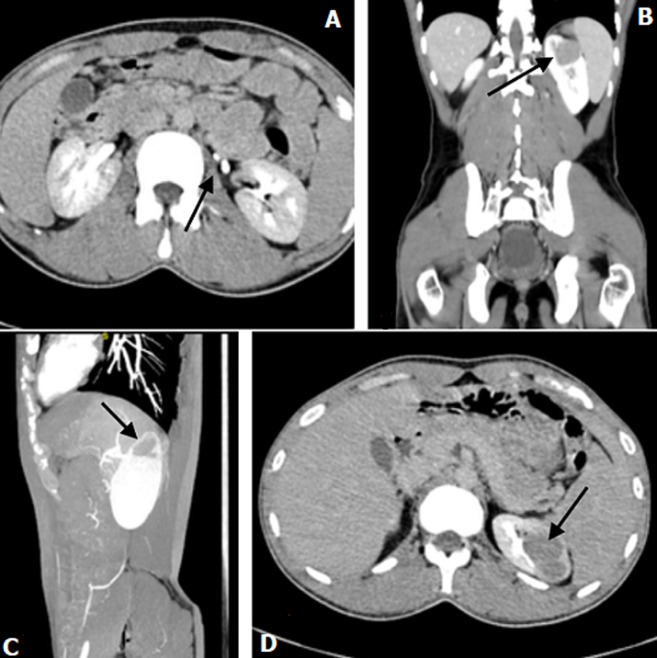
CT imaging of duplex kidney and renal tumor (arrow); axial view showing two ureters (arrow) (A); renal mass in the upper pole of the left kidney in coronal view (arrow) (B); in sagittal view (arrow) (C); in axial view (arrow) (D)

**Diagnostic assessment:** pre-operative blood investigations revealed Hb: 13 g/dl, WBC count: 7x10^3^/µl, Urea: 35mg/dL, creatinine: 1.4 mg/dL. Urine analysis revealed no microscopic hematuria or pus cells. The patient was subjected to computed tomography (CT) scan, which revealed a well-defined 5x4 cm exophytic lobulated heterogeneously enhancing soft-tissue attenuation mass lesion from the upper pole calyx of the left kidney suggestive of neoplastic etiology associated with duplex renal pelvis with bifid ureters on the left side. No evidence of venous thrombosis or metastasis to the local surrounding structure was noted (Figure 1).

**Figure 2:**
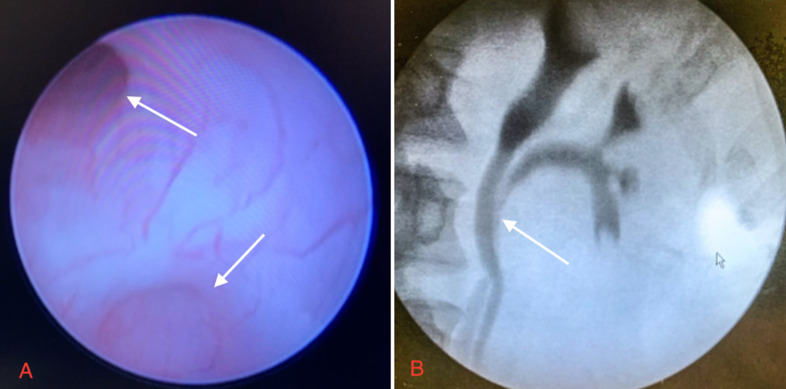
endoscopic view showing two ureteric openings (arrow) (A); fluoroscopic image showing the confluence of two ureters (arrow) (B)

**Figure 3:**
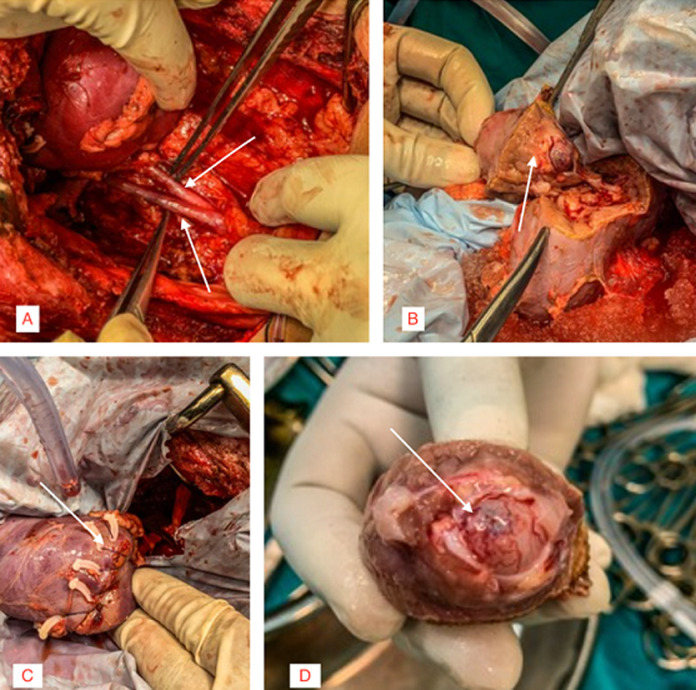
intraoperative findings of duplex system with renal tumor double ureter (arrow) (A); excision of the upper pole of left kidney along with tumor mass (B); meticulous suturing of open pelvicalyceal system (C); a wholly resected specimen of the tumor mass (D)

**Therapeutic intervention:** patient was planned for Surgical Intervention. After obtaining fitness from Anesthesia, the patient underwent ureteroscopy wherein the confluence of two ureters was identified. The ureteric stent was placed, and dye was injected, following which an x-ray was taken using C-Arm, which further confirmed the bifid ureter anomaly (Figure 2). With the patient in the right lateral position, the left flank incision was taken and 11^th^ rib was removed to reach the retroperitoneum with adequate exposure. Renal hilum was identified and bulldog clamps were applied over renal vein and artery. The kidney was covered with ice packs to prevent ischemia and bleeding. The incision was taken over the renal capsule. Partial nephrectomy with excision of tumor mass followed by meticulous suturing of the opened pelvicalyceal system over 6/6 Fr double j stent was performed (Figure 3). Bulldog clamps were released, hemostasis was achieved, and the specimen was sent for histopathological analysis.

**Follow-up and outcome:** the postoperative course in the hospital was uneventful. The histopathology report revealed a tumor mass of 5x3 cm in the upper pole of the left kidney. On the cut section, greyish white solid growth was noted. On microscopy, features of malignant epithelial cells with clear cytoplasm, acinar growth pattern, foci of dystrophic calcification and capsular invasion was noted suggesting of clear cell RCC with T1N0M0 stage. Sutures were removed and patient was discharged with a healthy scar line on postoperative day 11.

**Patient perspective:** the patient was happy with the decision of partial nephrectomy and the successful outcome of the surgery.

**Informed consent:** informed consent was obtained from the patient for participation in our study.

## Discussion

Duplex kidneys alone constitute 7.2% of CAKUT. Incidence of duplex kidneys is around 1% in a population of healthy individuals, and it is around 2-4% in a population of symptomatic individuals who have been explored, investigated, and diagnosed with various diseases of the urinary tract system [5,6]. This indicates that it is not an uncommon congenital kidney anomaly. However, the speculation of the incidence of this anomaly is much higher because most of these patients are asymptomatic for their lifetime. This leads to altered statistical data due to underreporting [7]. Most of these patients are asymptomatic, but they can land up into complications like vesicoureteral reflux, urolithiasis & obstruction at any time [8].

Due to advancements & improvements in recent technology, CAKUT can now be diagnosed during the prenatal assessment so that the management of the disease after delivery can be planned [2]. Based on the embryological development, this duplex kidney anomaly has been sub-categorized into two types: complete and incomplete duplication. Two independent urinary tracts are developed on the same side, with both ureters opening into the bladder separately in the complete duplication subtype. In contrast, in the case of incomplete duplication variety, two renal pelvises and ureters join together, forming a Y-shaped structure with a single opening into the bladder [9]. Routine ultrasonography of the abdomen can diagnose duplex kidneys. If they are asymptomatic, no further treatment is required for such patients. However, if there are any signs of complications leading to a non-functioning kidney, then excision of the non-functioning moiety of the kidney is usually advocated [10].

Kidney cancer accounts for approximately 2% of all cancer diagnoses and cancer deaths worldwide [3]. Aetiopathogenesis & incidence of coexistence of duplex kidneys and RCC are not well known due to the lack of literature in this corner of the study [11]. Kidney cancer is usually familiar in males and the old age group [3]. In our case, the patient was male and 28 years old. The major established risk factors for RCC include excessive body weight, hypertension, and cigarette smoking. Genetic factors also contribute to RCC risk, as evidenced by individuals with a family history of RCC having an approximately two-fold increased risk [3]. However, there was no evidence of any significant hereditary inheritance or established risk factors in our patient.

The most common presenting features of RCC include flank pain, gross hematuria, and a palpable abdominal mass. Nowadays, most diagnoses result from incidental findings due to the widespread use of non-invasive radiological techniques [3]. In our case, the patient presented with a chief complaint of abdominal pain for two months and hematuria for three days. CT with contrast study is ideal for diagnosing kidney tumors and staging the tumor. Characteristic features which will be noted are heterogenicity & exophytic growth of tumor [3]. CT Urography was done for our patient, which revealed a mass measuring 4x3 cm located in the upper pole cortex of the left kidney, suggesting neoplastic etiology with no radiological evidence of regional lymphadenopathy. However, CT stunned with an associated incidental finding of the duplex renal pelvis with bifid ureters arising from the ipsilateral kidney wherein ureters merged distally in the left iliac fossa to form a single ureter with a single vesicoureteric junction. The duplex kidney is defined as a renal unit comprised of two pelvicalyceal systems and is the most frequent congenital anomaly of the urinary tract. There are many variations within this condition. In our case patient had an incomplete duplex kidney (Bifid ureter variant). This coincidence of RCC with duplex kidneys is unique and is documented inconsequentially in English literature. Henceforth it was managed very vigilantly by skillful and experienced surgeons. Radical/partial nephrectomy by open/laparoscopic approach is the mainstay of treatment for RCC. Since the TNM stage of our patient was T1N0M0 and associated with congenital anomaly of the kidney, partial nephrectomy by open approach was planned. Intra-operatively after meticulous dissection, a bifid ureter was evidenced, correlating the radiological finding. A tumor was identified in the upper pole of the left kidney. A partial nephrectomy was performed, and the specimen was sent for histopathological diagnosis. Clear cell type of RCC was confirmed histopathologically. A similar study was done on a patient with duplex kidney and RCC by Mohan *et al*., wherein the right nephrectomy was performed in which the tumor mass measured 5.1x4.7x3.5 cm in the lower pole [2].

## Conclusion

The coexistence of a unilateral duplex kidney with incomplete ureteral duplication and ipsilateral renal cell carcinoma on the upper pole could be more than a coincidence. There is an immense lack of literature relating the duplex kidneys as an associated risk factor for renal malignancy. However, the underlying pathogenesis of this association with the possibility of a common genetic and molecular basis requires further analysis. Henceforth, this case report hopes to trigger the various international research institutes for detailed study and research in this direction, revealing a piece of more important hidden information regarding the pathogenesis of RCC at the genetic and molecular level.
